# Melanosomes in pigmented epithelia maintain eye lens transparency during zebrafish embryonic development

**DOI:** 10.1038/srep25046

**Published:** 2016-05-04

**Authors:** Masanari Takamiya, Feng Xu, Heikki Suhonen, Victor Gourain, Lixin Yang, Nga Yu Ho, Lukas Helfen, Anne Schröck, Christelle Etard, Clemens Grabher, Sepand Rastegar, Günther Schlunck, Thomas Reinhard, Tilo Baumbach, Uwe Strähle

**Affiliations:** 1 Institute of Toxicology and Genetics, Karlsruhe Institute of Technology (KIT), Postfach 3640, 76021 Karlsruhe, Germany; 2 Institute for Photon Science and Synchrotron Radiation (IPS), Karlsruhe Institute of Technology (KIT), 76021 Karlsruhe, Germany; 3 European Synchrotron Radiation Facility, 38043 Grenoble, France; 4 Eye Center, Freiburg University Medical Center, Killianstr. 5, 79106 Freiburg, Germany; 5 University of Helsinki, Department of Physics, 00560 Helsinki, Finland; 6 Department of Environmental Pollution and Health, State Key Laboratory of Environmental Criteria and Risk Assessment, Chinese Research Academy of Environmental Sciences, 100012 Beijing, China

## Abstract

Altered levels of trace elements are associated with increased oxidative stress that is eventually responsible for pathologic conditions. Oxidative stress has been proposed to be involved in eye diseases, including cataract formation. We visualized the distribution of metals and other trace elements in the eye of zebrafish embryos by micro X-ray fluorescence (μ-XRF) imaging. Many elements showed highest accumulation in the retinal pigment epithelium (RPE) of the zebrafish embryo. Knockdown of the zebrafish *brown* locus homologues *tyrp1a/b* eliminated accumulation of these elements in the RPE, indicating that they are bound by mature melanosomes. Furthermore, *albino* (*slc45a2*) mutants, which completely lack melanosomes, developed abnormal lens reflections similar to the congenital cataract caused by mutation of the myosin chaperon Unc45b, and an *in situ* spin trapping assay revealed increased oxidative stress in the lens of *albino* mutants. Finally transplanting a wildtype lens into an *albino* mutant background resulted in cataract formation. These data suggest that melanosomes in pigment epithelial cells protect the lens from oxidative stress during embryonic development, likely by buffering trace elements.

Trace elements are essential for normal development and physiology of the organism. These elements are found at the core of functional domains of metalloproteins in nearly all biological pathways. The presence of metal ion transporters with diverse metal affinities and their specific subcellular localization indicate that each metal needs to be actively transported into proper intracellular compartments. For example, certain transition metals (manganese, iron, copper, zinc) are required in mitochondria for aerobic respiration, heme synthesis and other functions. Mutation of the mitochondrial iron importer Slc25a37 (Solute carrier family 25 [mitochondrial iron transporter], member 37) causes hypochromic anaemia and erythroid maturation arrest in zebrafish^1^. In humans, mutations in the iron storage protein FTL (ferritin, light polypeptide) and the iron exporter SLC40A1 (solute carrier family 40 [iron-regulated transporter], member 1) lead to hyperferritinemia-cataract syndrome (OMIM #600886). Thus, proper transport and storage of metals play a crucial role in many physiological processes.

Aberrant localisation or accumulation of trace elements can lead to oxidative stress. The formation of hydroxyl radicals (·OH) from hydrogen peroxide (H_2_O_2_) is catalysed for example by metal ions such as iron and copper (the Fenton reaction)^2^. Tissues like the lens containing densely packed crystallins, which undergo little turnover over the entire lifespan of an organism^3^ are particular sensitive to oxidative stress. A proteomics study of the age-related cataractous lens identified modified amino acids in human crystallins, which led to their aggregation and caused light scattering^4^. Reactive oxygen species (ROS) such as superoxide (O_2_^.−^) and hydroxyl radicals (.OH) which are generated endogenously in living organisms have been considered as a cause of such deleterious post-translational modifications^5^. Interestingly, the severity of cataracts was reported to correlate with the amount of hydroxyl radicals in human lenses^6^. Detoxification of ROS and ROS-derived damage in the lens is merely achieved by enzymatic reduction of H_2_O_2_ (catalase and glutathione peroxidase) and the buffering of protein oxidation by antioxidants (i.e. ascorbic acid and reduced glutathione [GSH])^7^. To minimize light scattering, a great portion of the lens has lost organelles in the course of differentiation, including endosomal/lysosomal compartments that facilitate degradation of damaged proteins^8^. Another unique feature of the lens is its high permeability to ions, water, nutrients and other small molecules, due to ion pumps, channels and gap junctions. Age-related reduction of inward diffusion of reduced glutathione, together with the progressive loss of crystallin chaperone function, leaves the central part of the lens highly vulnerable to oxidative stress^9^.

To visualize sub-cellular element distributions in biological tissues, a number of chemical, or genetically encoded, fluorescent indicators have been developed for some elements such as calcium^10^ and zinc^11^. Although these fluorescent indicators have revealed dynamics of individual metal ions in many biological processes, the endogenous subcellular element distribution needs to be verified by direct visualization. In this context, hard X-ray fluorescence microscopy (μ-XRF) provides complementary but also unique information. Many elements of different chemistry and quantity can be correlatively analyzed for subcellular location and quantified with a dynamic range of more than 10,000:1. Although μ-XRF imaging has been applied to many different types of tissues, high resolution data on the localization of trace elements in the developing eye have been lacking. We studied zebrafish as a model organism due to its distinct advantages over other species. Its small size allowed us to scan the whole eye for mapping trace element distribution with high image resolution (100–300 nm). It also enabled us to examine the consequences of genetic mutations or gene knock-downs.

We report here the *in situ* element distribution in the eyes of zebrafish embryos. We found that mature melanocytes in the pigmented epithelial layer of the eye are major fixation loci for many elements. *albino* mutant embryos that lack melanosomes showed abnormal lens reflections. The cataractous lenses from *albino* mutants concurrently showed a signature of ectopic lens protein radicals caused by ROS. These results imply that melanosomes contribute to lens integrity by buffering elements that would otherwise cause deleterious lens protein modifications.

## Results

### Distinct chemical elements are predominantly enriched in the RPE

**Figure 1:**
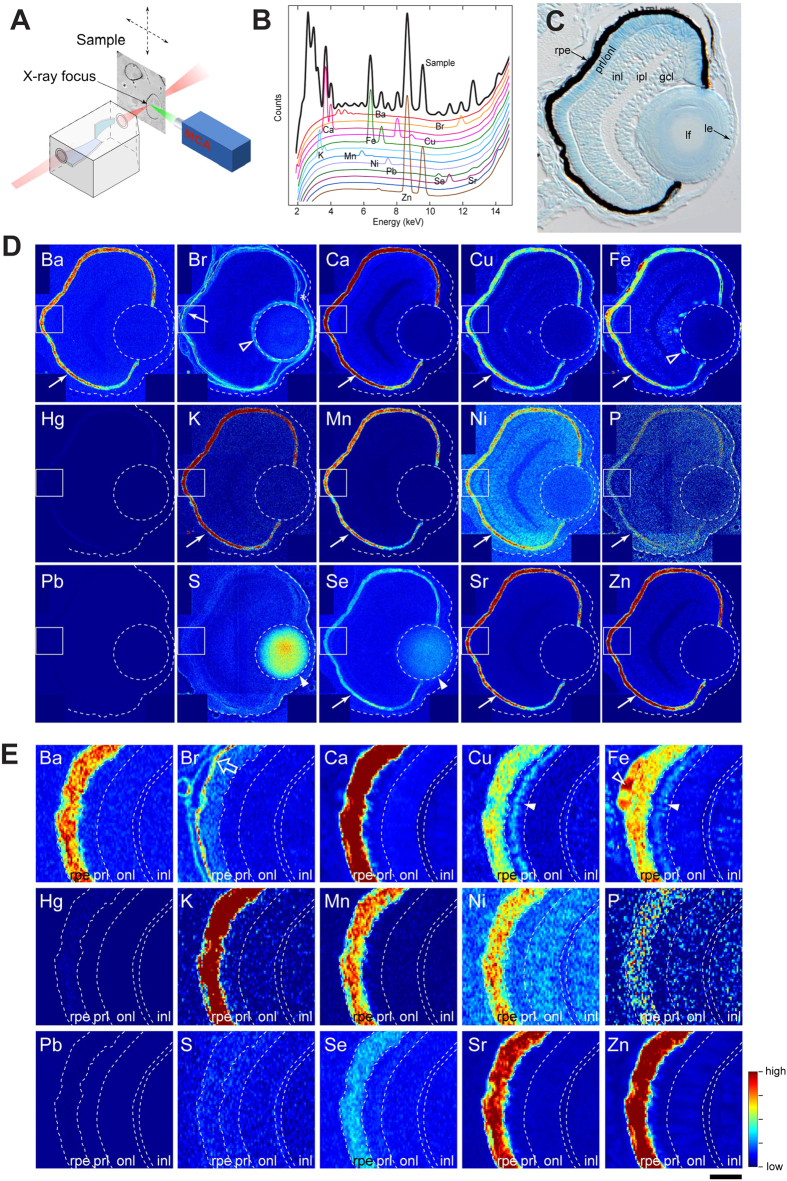
Distribution of inorganic elements in the eye of zebrafish embryos. (**A**) Illustration of μ-XRF imaging setup. The beam is generated in the synchrotron using an undulator source. The focusing optics is a Kirkpatrick-Baez (KB) mirror system, consisting of a pair of elliptically bent mirrors. The distance from the source to the KB is about 60 m, and from the KB to the focus about 0.18 m, allowing the incoming radiation to be focused into a sub-100 nm spot. The sample is scanned in the focal spot, and the fluorescence radiation is collected using an energy sensitive silicon drift diode and a multi channel analyser (MCA). (**B**) An example of a fluorescence spectrum recorded from the sample (black) overlaid with standard spectra from individual elements. Elements emit X-rays at energies that are characteristic to the given element, allowing the elements in the sample to be identified based on the peak locations. The spectrum shown here is a sum of spectrums from individual pixels covering about one quarter of the eye. (**C**) Toluidine blue-stained transverse 10-μm thick EPON section of 3-dpf zebrafish eye. rpe: retinal pigment epithelium, prl/onl: photo-receptor layer/outer nuclear layer, inl: inner nuclear layer, ipl: inner plexiform layer, gcl: ganglion cell layer, lf: lens fibre, le: lens epithelium. (**D**) Localization of elements in the eye of a 3-dpf embryo. Most elements are enriched in the RPE (arrows). Note that sulphur and selenium are also highly abundant in the lens fibre (arrow head). Stippled circle indicates the lens. The outline of the eye is given in a stippled line. Rectangular area denotes the region shown in the panel E. (**E**) Magnified view of the retinal area close to the RPE. The open arrow for Br points out the extracellular matrix. Step size: (**D,E**) 300 nm/pixel. Scale bars: (**C**) 26.5 μm (**D**) 30 μm; (**E**) 6 μm. Colour scale: Ba, 4–150 ppm; Br, 0–10 ppm; Ca, 10–3,000 ppm; Cu, 1–15 ppm; Fe, 2–80 ppm; Hg, 2–30 ppm; K, 20–300 ppm; Mn, 3–30 ppm; Ni, 0–7 ppm; P, 100,000–200,000 ppm; Pb, 0–200 ppm; S, 10,000–50,000 ppm; Se, 0–9 ppm; Sr, 1–50 ppm; Zn, 3–500 ppm.

We examined the element distribution in zebrafish embryonic eyes by X-ray fluorescence (μ-XRF) imaging (Fig. 1A). We first examined embryos at 3 days post-fertilization (dpf), when the basic eye structures have formed (Fig. 1C). Embryos were fixed, embedded in epoxy resin and sectioned at 10 μm thickness for μ-XRF imaging (Fig. 1A,B). The different eye tissues (Fig. 1C) were analyzed for quantitative distribution of the following 15 elements: Ba, Br, Ca, Cu, Fe, Hg, K, Mn, Ni, P, Pb, S, Se, Sr and Zn. Mercury and lead were barely detected (Fig. 1D). For the remaining 11 elements except sulphur and bromine (Ba, Ca, Cu, Fe, K, Mn, Ni, P, Se, Sr and Zn), the highest concentration was found in the RPE (filled arrows in Fig. 1D,E; Table 1). Sulphur showed strong enrichment in the lens nucleus (Fig. 1D, filled arrowheads). Selenium was also enriched in the lens, albeit at concentrations lower than those in the RPE (Fig. 1D, filled arrowheads). Bromine exhibited a distinct distribution and was detected in the basement membrane regions of the RPE (arrow, Fig. 1D; open arrow Fig. 1E), lens epithelium (open triangle, Fig. 1D) and corneal endothelium (asterisk, Fig. 1D). An enhanced iron signal was detected in the pigment epithelial layer (arrow, Fig. 1D) as well as in the highly vascularised choroidal and primordial hyaloid vasculature (open triangle, Fig. 1D). In the latter, iron contained in heme of red blood cells may likely contribute to the signal (open triangle, Fig. 1D,E). The photoreceptor layer of the retina contains higher amounts of copper and iron (filled triangles, Fig. 1E) than the other inner retinal layers. In light of these observations, we focused on the accumulation of elements in the RPE, which suggests the presence of a dedicated storage structure with potential biological function.

### Mature melanosomes are required for enrichment of inorganic elements in the RPE

**Figure 2:**
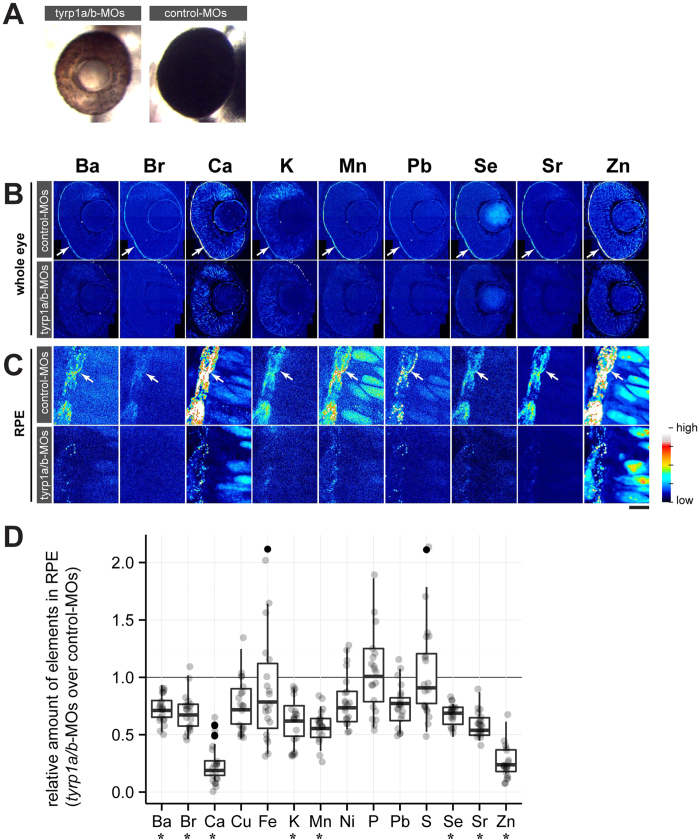
Mature melanosomes are required for enrichment of inorganic elements in the RPE. (**A**) Morpholino oligonucleotide (MO)-mediated knockdown of Tyrp1a/b results in the absence of mature melanosomes (brown colour of RPE) at 2 dpf, while control-MO injected embryos form mature melanosomes (black colour of RPE). (**B**) Distribution of inorganic elements in the eye of 2 dpf embryos injected with control (*control-MOs*, upper row) or *tyrp1a/b*-MOs (*tyrp1a/b-MOs*, lower row). The RPE enrichment of elements (arrow) seen in control-MO injected embryos is not observed in *tyrp1a/b*-MO injected embryos (*n* = 3 for each group). Step size: 300 nm/pixel. (**C**) Images taken with high lateral resolution (100 nm/pixel) showing inorganic element enrichment in individual melanosomes in the RPE (arrow) of embryos injected with control-MOs (upper row). This enrichment is not evident or strongly reduced in *tyrp1a/b*-knockdown embryos (lower row). Colour scale: Ba 10.0–132.5 ppm; Br 0.1–6.5 ppm; Ca 0–1152 ppm; K 0–623.3 ppm; Mn 0.3–19.6 ppm; Pb 0.7–10.8 ppm; Se 0.3–4.6 ppm; Sr 0.3–19.6 ppm; Zn 0–152.9 ppm. Scale bars: (**A,B**) 50 μm; (**C**) 5 μm. (**D**) The content of each element in the RPE was shown as the ratio of *tyrp1a/b* morphants to control embryos, with all possible combinations between control MO-injected embryos (*n* = 4 embryos) and *tyrp1a/b* MO-injected (*n* = 5 embryos). Abundance of eight elements (Ba, Br, Ca, K, Mn, Se, Sr and Zn) showed significant reduction in the RPE after knockdown of *tyrp1a/b* (Mann–Whitney *U* test; *U* = 0, *p = 0.0158 for Ba, Ca, K, Mn, Sr and Zn; *U* = 1, *p = 0.0365 for Br; *U* = 0, *p = 0.0194 for Se).

To examine whether melanosomes are responsible for the observed enrichment of elements in the pigment epithelial layer, we analyzed the distribution of these elements after morpholino oligonucleotide (MO)-mediated knockdown of the two zebrafish homologues of the mouse *brown* locus protein, *tyrosinase-related protein 1a* and *b* (*tyrp1a/b*). Tyrp1 expression in melanocytes correlates with the onset of melanin biosynthesis^12^. Previous studies showed severe melanosome defects in morphants generated by knock-down of Tyrp1a/b, with an arrest of melanosome maturation in the RPE at the stage II premelanosome^13^, leading to the formation of brown pigments instead of the black melanin (Fig. 2A). Upon knock-down of Tyrp1a/b, we found a significant reduction of elements localized in the RPE at 2 dpf (Mann-Whitney *U* test; *p* = 0.0158 for Ba, Ca, K, Mn, Sr and Zn; *p* = 0.0365 for Br; *p* = 0.0194 for Se; Fig. 2B,D and Table 2), compared with control-MO-injected embryos (upper rows of Fig. 2B,C). High resolution imaging with a 100 nm-step size revealed localization of many elements to individual subcellular structures compatible with melanosomes in the control-MO injected groups (white arrows, Fig. 2C upper row; 200–500 nm in diameter). This signal distribution pattern was lost in *tyrp1a/b* morphants, which are characterized by melanosome maturation arrest at the premelanosomal stage (Fig. 2C, lower row). Pigment epithelial cells are known to express enzymes that bind metal ions, such as catalase (Fe), cytoplasmic superoxide dismutase (Cu, Zn) and mitochondrial superoxide dismutase (Mn)^14^,^15^. Furthermore, our results suggest that melanin itself may be a biological material responsible for fixation of barium, bromide, calcium, potassium, manganese, selenium, strontium and zinc.

### Lack of pigment epithelial melanosomes sensitizes the lens to cataract formation

To explore the physiological role of inorganic element accumulation in pigment epithelial melanosomes during embryonic development (Fig. 2B–D), we focused on the lens, hypothesizing that defective metal accumulation in the RPE may increase the risk of cataract formation. We adopted confocal reflection microscopy to allow for quantitative assessment of cataract formation *in vivo*. This labelling-free non-invasive imaging method visualises contrast created at the structural boundaries of different refractive indices^16^. To validate the feasibility of this method for early cataract detection, we examined homozygous zebrafish *steif* mutants, which carry a loss-of-function allele of the myosin chaperone Unc45b^17^. In humans, UNC45B plays an essential role in maturation of the lens fibre cells and its mutation causes congenital cataract^18^, reflecting the importance of lenticular myosin arranged into polygonal arrays at the apical cytoplasm of the lens epithelium^19^. Confocal reflection imaging revealed significantly abnormal lens reflections arranged in concentric circles in *unc45b* mutants at 4 dpf, clearly indicating cataract formation (Welch two sample *t*-test *p* = 2.61 × 10^−11^, Fig. S1A–E). Although *unc45b* mutants have been shown previously to have smaller eyes and ectopic cell nuclei in the lens^18^, congenital cataract formation in the absence of Unc45b protein has not yet been demonstrated in zebrafish.

**Figure 3:**
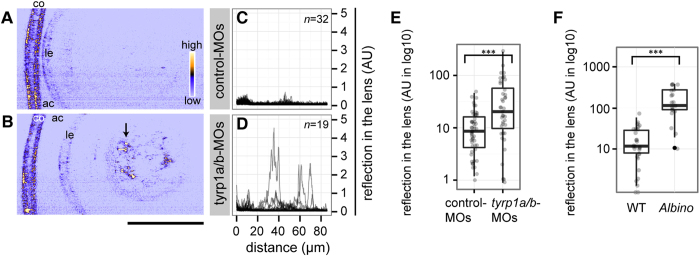
Absence of mature melanosomes sensitizes the lens fibre to cataract formation. (**A**,**B**) Confocal reflection imaging on the lenses at 4 dpf of living embryos injected with either control-MOs (**A**) or *tyrp1a/b*-MOs (**B**). The anterior chamber (ac) is oriented to the left. co: cornea. le: lens epithelium. Scale bar: 50 μm. The arrow in panel B points out abnormal lens reflection observed after *tyrp1a/b*-knockdown. (**C**,**D**) Lenticular reflection profiles as a function of distance from the anterior edge of the lens capsule (lc) toward the posterior end of the lens for control-MO injected embryos (**C**) and *tyrp1a/b*-MO injected embryos (**D**). The intensity of reflection is shown in an arbitrary unit (AU). The number of examined individuals for each group is shown in the upper right corner. Profiles from the individual embryos were overlaid. (**E**,**F**) Quantification of abnormal lens reflections. Welch two sample *t*-test and Mann-Whitney *U* test showed significant differences between control-MOs (*n* = 51 embryos) and *tyrp1a/b*-MO injections (*n* = 40 embryos; (**E**) ***p = 8.74 × 10^−4^, *t* = 3.5896), and between wild type embryos (WT, *n* = 29 embryos) and *albino* (*slc45a2*) homozygous embryos (*n* = 19 embryos; (**F**) ***p = 1.52 × 10^−7^, *U* = 26), respectively.

We next examined whether impaired melanosome biogenesis would affect the lens. First we analysed the lenses of *tyrp1a/b*-knockdown embryos with immature brown pigments at 4 dpf (Fig. 2A). Embryos injected with antisense morpholinos were raised in the dark and the lens was examined at 4 dpf. Tyrp1a/b-knockdown embryos (Fig. 3B,D) exhibited abnormal lens reflection values in comparison to control-MO-injected embryos (Fig. 3A–E; *p* = 8.74 × 10^−4^, Welch two-sample *t*-test).

To further examine whether melanosomes might protect against cataract formation, we examined a melanosome mutant, *albino* (*alb*^*b4/b4*^, *slc45a2*). Homozygous *albino* mutants completely lack melanosomes in the RPE with no obvious defects in the other two types of pigment cell types (xanthophores and iridophores)^20^,^21^. As in *tyrp1a/b* morphants, we found a significantly increased lens reflection in *albino* mutants at 4 dpf grown in the dark (Fig. 3F; *p* = 1.52 × 10^−7^, Mann-Whitney *U*-test). Thus, evidence from two impaired melanosome biogenesis phenotypes supports the hypothesis that mature melanosomes protect against cataract formation during embryonic development.

### Cataractous lenses in albino mutants contain increased levels of ROS-modified proteins

**Figure 4:**
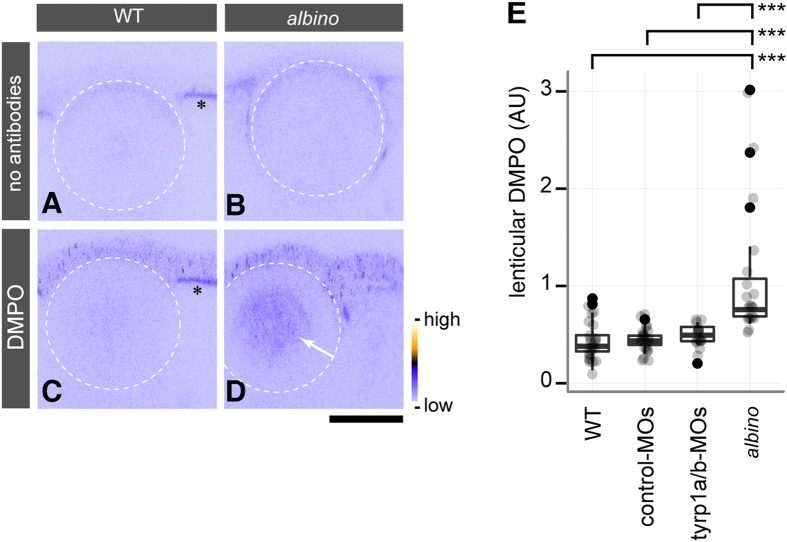
The cataractous lens in *albino* mutants shows lens protein radicals caused by oxidative stress. (**A–D**) *In situ* DMPO adduct distribution in the eye of wild type (WT) (**A**,**C**) and *albino* homozygous mutants (**B**,**D**) at 4 dpf. The white stippled circles outline the lens. *albino* homozygous mutants show abnormal DMPO adducts in the nucleus of the lens (**D**, arrow), visualised by staining with an anti-DMPO antibody and a fluorescent secondary antibody, whereas this staining is absent from the lens of WT embryos (**C**). The panels (A,B) show images obtained with the staining solutions lacking antibodies for WT and *albino* embryos, respectively. Asterisks (**A**,**C**) indicate the autofluorescence observed in the RPE of WT. The colour ramp next to panel (D) denotes the fluorescent signal intensity for DMPO adducts. Scale bars: 50 μm. (**E**) Quantification of DMPO adducts in the lens. One-way ANOVA showed significant differences (*F*[3, 78] = 15.674, *p* = 4.54 × 10^−8^) between *albino* homozygous mutants (*n* = 19 embryos), un-injected WT (*n* = 24 embryos), and WT embryos injected with control-MOs (*n* = 22 embryos) or *tyrp1a/b*-MOs (*n* = 17 embryos). A significant increase of DMPO adducts was observed in the lenses of *albino* mutants in comparison to all other conditions (Tukey HSD test; ***p = 3.0 × 10^−7^ for WT vs. *albino*; ***p = 8.0 × 10^−7^ for control MO-injected WT vs. *albino*; ***p = 2.02 × 10^−5^ for *tyrp1a/b* MO-injected WT vs. *albino*).

ROS such as hydroxyl radicals (HO·) produced in the lens are suggested to cause irreversible modifications of lenticular proteins that eventually transform them into insoluble forms^6^,^22^. To gain further insight into the mechanisms of cataract formation in the embryos with impaired pigmentation, the lenses from wildtype and homozygous *albino* mutants were examined for the presence of oxidative stress by a spin trapping immunoassay using 5,5-dimethyl-1-pyrroline-*N*-oxide (DMPO) labelling to detect macromolecule radicals generated by oxidative stress^23^,^24^. Groups of wildtype and *albino* mutant embryos were kept in fish water in the dark in the presence of 200 mM DMPO from 24- to 96 hpf and were then processed for immunohistochemistry with an anti-DMPO antibody. Wildtype embryos showed staining levels in the lens comparable to background values determined in a negative staining control without antibodies (Fig. 4A,C). In contrast, homozygous *albino* mutants showed increased staining of DMPO adducts in the lens (Fig. 4D arrow; one-way ANOVA/TukeyHSD test, *p* < 0.001, Fig. 4E). We failed to observe such a significant increase in the brown pigment containing *tyrp1a/b* morphants in comparison to embryos injected with control-MOs (one-way ANOVA/TukeyHSD test *p* = 0.970; Fig. 4E). Technical difficulties to achieve complete gene knockdown at this late stage of development and low staining sensitivity may have contributed to this finding (Fig. 4E).

### Lens transplantation demonstrates lens non-autonomous effect on cataract formation in albino mutants

**Figure 5:**
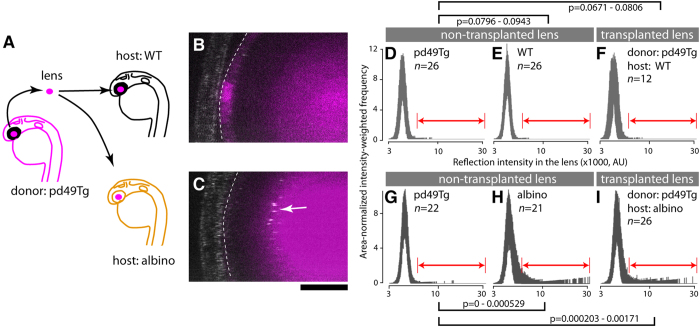
A wildtype lens develops cataract when transplanted into a *slc45a2* (*albino*) mutant background. (**A**) Lens transplantation scheme. The lens derived from a transgenic wildtype line *pd49Tg* was used as a donor allowing identification of the *slc45a2*+/+ lens by expression of RFP after transplantation into mutant (*albino*) and wildtype (WT) host embryos. Lens transplantation was performed among stage matched donor and host embryos during 26–30 hpf. The endogenous host lens was completely removed and replaced with *pd49Tg* donor lens. Embryos transplanted with *pd49Tg* donor lenses were raised in the dark and the presence of abnormal lens reflection was examined at 4 dpf. (**B**,**C**) The stippled line demarcates the transplanted *pd49Tg* donor lens identified by the presence of RFP fluorescence reporter (magenta). The reflection channel is merged in gray. Note the presence of abnormal lens reflection in the transplanted lens when host embryos are *slc45a2/albino* mutants. The anterior chamber is oriented to the left. Scale bar: 20 μm. (**D**–**I**) Reflection signal distribution in the lens of each group is shown as a function of the reflection intensity value (x-axis; x1000, log10 scale in AU) with intensity-weighted frequency (y-axis; the product of intensity and frequency, normalized for area). The number of embryos for each group is given in each panel. Results are shown as a set of two independent experiments (**D**–**F** and **G**–**I**), each composed of donor *pd49Tg* non-transplanted lens (**D**,**G**), non-transplanted lens in the host (wildtype [**E**] and *albino* [**H**]), and the transplanted donor *pd49Tg* lens in WT host (**F**) and *albino* host (**I**). A range of reflection signals indicated by double-headed arrows (red) were considered as abnormal and used for permutation tests. 99% confidence interval of *p*-values is given for each indicated pair.

Next, we wished to exclude that the formation of cataract were caused by lack of *slc45a2* (*albino*) gene function in the lens. We thus performed lens transplantation experiments (Fig. 5A). The donor lens was derived from a transgenic wildtype line *pd49Tg* to facilitate detection of the transplanted lens in the eye of *slc45a2* (*albino*) mutant and wildtype hosts. The lens transplantation was performed between stage-matched donor and host embryos at 26–30 hpf in two sets of experiments. One lens of the host was completely removed and replaced with a *pd49Tg* donor lens. Operated embryos were raised in the dark and the presence of abnormal lens reflection was examined at 4 dpf. The *pd49Tg* donor lens did not show significant lens abnormality in comparison to those of non-transgenic wild type embryos (permutation test *p*-values = 0.0796–0.0943; 99% confidence interval is given hereafter). Transplanted *pd49Tg* lenses into wildtype host embryos showed comparable lens quality to non-transplanted *pd49Tg* siblings (permutation test *p*-values = 0.0671–0.0806; Fig. 5B; compare D and F), indicating that the transplantation itself did not significantly cause abnormal lens reflection. In contrast, transplantation of *pd49Tg* lenses into *albino* hosts caused significant increase of lens reflection (permutation test *p*-values = 0.000203–0.00171; Fig. 5C; compare G and I). Thus, the transplanted lens with the wildtype *slc45a2* allele displayed the mutant cataract phenotype in *albino slc45a2* mutants. These results suggest a lens non-autonomous function of *slc45a2* (*albino*). This is consistent with the notion that melanosomes have a role in protecting the lens from cataract.

## Discussion

We analyzed trace element distribution in the zebrafish eye with sub-micrometer image resolution and found that many elements are highly enriched in the pigment epithelial (PE) layer. Intervening with melanin biosynthesis and mature melanosome formation significantly reduced detected element levels and enrichment in the PE, implying mature melanosomes as primary sequestration loci for various elements. Moreover, melanosomes appear to protect the lens from cataract formation: Elimination of melanosomes by inactivation of two different genes (*slc45a2* [*albino*]*, tyrp1a/b*) lead to cataract formation. The lenses of *slc45a* (*albino*) mutants showed signs of increased oxidative stress. Transplantations of wildtype lenses result in cataractous changes in transplanted lenses in *slc45a2* (*albino)* mutant but not in wild type hosts. Thus, melanosomes appear to protect the lens from cataract formation. We propose that binding of trace elements in melonosomes plays a buffering role which protects the lens from oxidative damage and cataract formation.

A major advantage of μ-XRF imaging is the direct observation of elements, independent of chemical indicators. We found a large number of trace elements enriched in the mature melanosomes in the RPE. Although it is not clear how the trace elements are fixed to melanosomes, a metal binding property of melanin in the eye has been well established at least for the following 23 elements: Ag^25^, Al^25^, Ca^25^,^26^,^27^, Cr^25^, Cs^25^, Cu^25^,^28^,^29^, Fe 25,26,30, Ga^25^, Hg^25^, K^25^,^27^, Mg^27^, Mn^25^,^30^,^31^, Rb^25^, S^26^, Sr^25^, Ni^25^, Pb^25^, Pd^25^, Pt^25^, Ti^25^, Tl^25^, V^25^, Zn^25^,^27^,^28^,^29^. Previously, zinc ion distribution has been studied using different fluorescent indicators, revealing a punctate cytoplasmic distribution in mouse fibroblasts^11^ as well as its localization to the endoplasmic reticulum and mitochondria in HeLa cells^32^. Although no nuclear signal has been detected in this previous study, our μ-XRF images showed zinc signals in the nuclei of retinal cells, in addition to a punctate cytoplasmic signal (Fig. 2C). Irregular subcellular distribution of chemical indicators, signal quenching in certain intracellular microenvironments or differences in sample preparation may account for these diverse observations. Furthermore, chemical indicators mainly report the presence of uncomplexed free metal ions in living cells, accounting for a minor proportion of total cellular metal content, e.g. less than 1% in case of iron^33^. On the other hand, our μ-XRF imaging was performed on epoxy-embedded fixed tissues. Although this limits the detection of metals to those tightly bound to proteins or other molecules, it visualizes the great majority of cellular metals.

Several elements showed a unique distribution pattern. For example, bromine specifically localized to basement membranes in the cornea, lens, retina, brain and epidermis, in addition to melanosomes (Fig. 1D). As another example of unique distribution patterns, sulphur and selenium localized to lens fibres (Fig. 1D). Considering that innate defensive mechanisms in the lens centre on redox homeostasis, it is reasonable to speculate that lenticular sulphur and selenium accumulation reflect the presence of glutathione and glutathione reductase, which contain sulphur and selenium, respectively. Aberrant exposure of female zebrafish to selenium leads to detrimental enrichment of selenium in the lens of the offspring underscoring the tight balance between beneficial and toxic levels of selenium^34^. Copper and iron were enriched in the photoreceptor layer (Fig. 1E, filled arrowheads). In adult rat retina, iron has been detected in photoreceptor cells in a proton-induced X-ray emission study^35^, thus supporting our observations.

Cataract formation is influenced by multiple factors such as genetics, age and environment. As the causative genes for cataract include genes involved in gap junctions (Gap Junction Protein, Alpha 3, 46 kDa [GJA3, OMIM #601885] and Gap Junction Protein, Alpha 8, 50 kDa [GJA8, OMIM #116200]), maintenance of permeability to small molecules appears as a key feature for lens transparency. This may leave the lens quite susceptible to ion composition changes in the vitreous body, including changes in metal ion levels. Accordingly, mutations in the iron storage protein FTL (ferritin light polypeptide) and the iron exporter SLC40A1 (solute carrier family 40 [iron-regulated transporter] member 1) are linked with cataract in the disease phenotype of hyperferritinemia-cataract syndrome (OMIM #600886), most probably as a result of increased radical production catalyzed by iron. Our spin trapping immunoassay results thus imply that zebrafish pigment mutants, in which we observed reduced trace element buffering, may experience elevated intraocular free metal ion concentrations. Free or labile metal ions are a source of ROS implicated in human eye disease^36^,^37^.

We could not directly prove the presence of increased metal ions in the lens of melanosome deficient embryos. Free metals may escape fixation. However, the correlative evidence is intriguing: Lack of melanosomes leads to loss of trace element binding in the RPE and concomitant increase of oxidative stress and cataract in the lens. The lens transplantation experiments clearly show that the primary cause of the cataract formation does not reside in the lens tissue itself but is the result of factors from the surrounding mutant tissue acting on the lens, - presumably ROS generating metal ions that cannot be buffered in the mutant melanosome-free environment. To further explore the role of melanosomes in the prevention of cataract formation, the impact of the lens opacity that we observed in *albino* homozygous embryos needs to be followed into later life, together with a study on how detoxification pathways (notably those regarding redox homeostasis) develop in the zebrafish lens. The contribution of RPE to lens clarity likely diminishes with time as the eye grows and diffusion distances increase. It is very likely that melanosomes of the later developing iris but also skin melanocytes bind trace elements in the same way as was measured here for the melanosomes of the RPE. In fact, preliminary data suggest that trace elements are also highly enriched in melanocytes residing in the skin close to the eye (unpubl.). It was demonstrated in adult mice that iris melanocytes possess a high ability to synthesize melanin, while the RPE showed only low melanin synthesis^38^. Considering that in the adult the pigmented tissue in the vicinity of the lens is not the RPE but the iris, the source of melanosomes as a metal buffer could be different in the adult from embryonic stages. As melanin itself was shown to change its metal binding properties with age^27^, further μ-XRF imaging of the adult eye will provide valuable information on the role of ocular melanosomes in maintaining lens transparency. Human oculocutaneous albinism (OCA) is frequently associated with reduced vision due to macular hypoplasia, refractive errors, nystagmus, strabismus and photophobia, whereas congenital cataracts are not a prominent clinical feature^39^. A necessity for early cataract surgery has been reported in some patients diagnosed with Hermansky-Pudlak syndrome, a distinct form of autosomal recessive tyrosinase-positive OCA^40^. In light of the significant macular, refractive and strabismological alterations in human OCA, mild forms of concomitant lens affections are likely underreported as they may often lack clinical relevance at the time of diagnosis. Alternatively, *albino* individuals may have developed a compensatory mechanism for increased ocular oxidative stress due to the lack of PE. Albino guinea pigs showed increased glutathione peroxidase activities in the retina in comparison to pigmented animals^41^. The increased cataract here observed with zebrafish *albino* embryos could be related to their development outside of the maternal body, being more susceptible to oxidative stress than mammalian counterparts in a controlled environment.

In summary, our data provide novel evidence for an antioxidant, protective effect of pigment epithelial cells *in vivo* and demonstrate a functional coupling of mature melanosomes in PE cells to homeostasis in adjacent tissues. Mature melanosomes may function as ion scavengers to prevent uncontrolled chemical interactions. A PE-mediated melanosome-dependent protective effect most likely also has implications for eye diseases other than cataract such as age-related macular degeneration (AMD), which is associated with accumulation of aberrant melanoliposomes and RPE cell loss. These observations warrant further characterization of melanosome function and ocular trace element distribution in eye disease.

## Methods

### Ethics statement

All zebrafish husbandry and experimental procedures were performed in accordance with the German animal protection standards and were approved by the Government of Baden-Württemberg, Regierungspräsidium Karlsruhe, Germany (Aktenzeichen 35–9185.64).

### Fish, preparation of samples for μ-XRF imaging

Fish were maintained at 28 ^o^C as previously described^42^. Zebrafish (*Danio rerio*) of the AB_2_O_2_ wildtype strain, as well as *unc45b*^*sb60/sb6017*^, *pd49Tg* and *albino* (*alb*^*b4/b4*^, *slc45a2*) were used in this study. For μ-XRF imaging the embryos were fixed at the 72 hpf stage with 4% paraformaldehyde/phosphate buffer saline, dehydrated in a graded series (50%, 75%, 90%, 95%, 100%) of ethanol, followed by propylene oxide immersion, and embedded in EPON 812 (glycid ether 100; Serva, Heidelberg, Germany), as described^43^. Polymerization was processed at 65 ^o^C for 16 hours. Thick 10 μm sections were cut with a microtome with a glass knife and recovered on 500 nm thick silicon-nitride (Si_3_N_4_) films.

### Morpholinos

To knockdown the expression of *tyrosinase-related protein 1a/b*, combinations of two antisense morpholinos reported previously^13^ were injected. To verify the sequence specificity, we used two antisense morpholinos with scrambled sequence. Morpholinos were synthesized by Gene Tools, LLC (Philomath, OR). tyrp1a_e1i1: 5′-ATCGGCCACAGTCACTTACCCACGG-3′; tyrp1b_atg: 5′-GCACTAAACACACACTCTTCCACAT-3′, control morpholino1: 5′-GAATAAGTCAGCTCTTCTCGCCAT-3′; control morpholino2: 5′-GGCCATTGCCTTAAGCTAATCAATA-3′.

### Element analysis of fish water and metal solutions

Fish water was prepared as 0.006% (w/v) instant ocean® aquarium sea salt dissolved in Milli-Q water (Millipore, Schwalbach, Germany). The samples were diluted by 1:10 and 1:100 with 2% HNO_3_ solution and measured by inductively coupled plasma mass spectrometry (ICP-MS, ELAN 6000; PerkinElmer, Rodgau-Jügesheim, Germany; Table S1) in a semi-quantitative mode.

### Immuno spin trapping assay with DMPO

DMPO and the anti-DMPO monoclonal antibody were obtained from Enzo Life Sciences (Cat No. ALX-430-090-M500 and ALX-803-340-C100, respectively). Groups of embryos, each composed of 20 embryos, were kept in 500 μl fish water with or without metal toxicants in the presence of 200 mM DMPO from 24- to 96 hpf. The DMPO concentration was determined to achieve a balance between tissue penetration and non-toxicity^24^. Embryos were fixed at 4 dpf with 4% paraformaldehyde/phosphate buffer saline (PBS) and the trunk parts of the embryos were removed manually to facilitate the following immuno-detection. The anti-DMPO antibody was used at 1:500 dilution with PBS/5% dimethylsulfoxide/0.7% TritonX/1% bovine serum albumin. Alexa Fluor 680 goat anti-mouse IgG antibody (1:1000 dilution; Life Technologies, Darmstadt, Germany) was used as a secondary antibody.

### Confocal reflection microscopy

Living embryos were anaesthetized by 0.0168% (w/v) MESAB (tricane methanesulfonate, MS-222; Sigma-Aldrich, Taufkirchen, Germany) and embedded into 0.5% (w/v) low melting agarose with the lateral side of the embryo facing toward the objective. A TCS SP5 confocal system with upright microscopy (DM6000; Leica Microsystems, Wetzlar, Germany) was used for visualization of the abnormal light reflection in the lens with a 63× water immersion objective (HCX APO L U-V-I 63.0 × 0.90 WATER UV). An acousto-optical beam splitter (AOBS) was set for reflection mode. Images were acquired in xzy scanning mode with a bit-depth of 8-or 16-bit. Three wavelengths (458, 476 and 488 nm) of light from an argon laser source were used as incident light to detect reflection (453–500 nm) with pinhole opening set to 0.75 airy unit.

### μ-XRF imaging

The samples were imaged at the European Synchrotron Radiation Facility (ESRF) nanoimaging endstation ID22NI (later moved to ID16B). An X-ray beam at 17 keV energy focused to an intense spot (60 nm × 80 nm FWHM, ~10^12^ phot/s) was used to raster scan the sample (Fig. 1A). The sample emits characteristic X-rays whose energies depend on the atom species inside the illuminated volume, and these are collected with a silicon drift diode detector (energy resolution full width at half maximum ~200 eV) to give a spectrum for each sample position (Fig. 1B). Scans with a step size of 300 to 500 nm were used to cover the whole eye, and scans with a 100 nm step size were performed in chosen regions of interest in the RPE. To avoid effects of radiation damage in the results, the high and medium resolution scans were done either on the two eyes on the opposite sides of the head or on the same eye on adjacent sections. For the data analysis, the mass fractions were quantified in each pixel using the program PyMCA^44^ and a calibration sample (NIST Standard reference material 1577b). Seven different eye anatomical regions shown in Fig. 1C were manually segmented and the average concentrations of the elements were calculated.

### Image analysis

μXRF and confocal fluorescent images in either 8-bit or 16-bit gray-intensity value were analysed with ImageJ^45^. For the quantification of lens reflection, a rectangular-shaped region-of-interest (ROI) with a defined size was set to cover the whole central region of the lens to obtain frequency distribution of gray levels within the ROI. The gray value distribution from individual images was collected on a spreadsheet for the calculation of frequency-weighted intensity values as the product of gray-intensity value in reflection channel and the number of occurrences in a ROI. The frequency-weighted intensity values were further normalized for area to obtain frequency-weighted intensity values per pixel area. Abnormal reflection signal in the lens of each embryo was calculated as the sum of area-normalized frequency-weighted intensity values with intensities higher than an arbitrary threshold, which was defined based on the wild type lens.

### Lens transplantation

Lens transplantation was performed among stage matched donor *pd49Tg* and host embryos (wildtype or *albino*) during 26–30 hpf according to the procedure^46^. Briefly, both donor and host embryos were pre-treated with calcium-free zebrafish Ringer’s solution (ZFR; 116 mM NaCl, 2.9 mM KCl, 5 mM HEPES pH7.2)^42^ for 5 minutes and further with 5 mM EDTA in calcium-free ZFR for additional 20 minutes. Using a plastic Petri dish (ϕ 6 cm), embryos were embedded laterally in 0.6% low melting point -agarose prepared in calcium-free ZFR containing an anaesthetic MS-222 (CAS# 886-86-2, Sigma-Aldrich). After solidification of the agarose, 0.2% agarose was overlaid to prevent the lens from adhering to the tungsten needle (ϕ 0.125 mm; Cat#10130-05, Fine Science Tools). The tip of the tungsten needle was electrically sharpened in 1N NaOH solution. Under a conventional binocular microscope, the donor and host lens was removed through a short incision in the cornea, using a fine tungsten needle held by hand. The transfer of the lens and insertion into the optic cup of an host embryo was conducted as described^46^. After transplantation, embryos were overlaid with ZFR (116 mM NaCl, 2.9 mM KCl, 1.8 mM CaCl_2_, 5 mM HEPES, pH7.2) for 30 minutes and released from agarose into zebrafish E3 medium^42^.

### Statistical analysis

All statistical tests were conducted with R^47^. Before comparing mean values between populations, each dataset with equal to or more than 15 biological replicates was tested for normality by Shapiro-Wilk test at an alpha level of 0.05. Datasets with normal distribution were subjected to two-tailed analysis with either Welch two sample *t*-test, or one-way analysis of variance (ANOVA) followed by Tukey’s honestly significant difference (HSD) test on-demand. For datasets with less than 15 embryos or non-normal distribution, either two-tailed analysis with Mann-Whitney *U* test or permutation test^48^ was chosen.

## Additional Information

**How to cite this article**: Takamiya, M. *et al.* Melanosomes in pigmented epithelia maintain eye lens transparency during zebrafish embryonic development. *Sci. Rep.* **6**, 25046; doi: 10.1038/srep25046 (2016).

## Supplementary Material

Supplementary Information

## Figures and Tables

**Table 1:** Quantification of elements in the zebrafish eye.

Elements	RPE	ONL/PR	INL	IPL	GCL	LF	LE
Ba	87.96 (11.72)	22.7 (1.61)	21.88 (1.45)	20.17 (0.74)	23.11 (3.18)	21.01 (1.12)	22.28 (1.37)
Br	2.2 (0.22)	0.63 (0.2)	0.55 (0.15)	0.58 (0.12)	0.64 (0.16)	0.84 (0.43)	1.47 (0.36)
Ca	3,053.85 (541.4)	281.89 (69.93)	198.6 (54.37)	76.44 (8.53)	152.66 (40.04)	96.03 (18.59)	204.48 (39.37)
Cu	6.43 (0.68)	2.02 (0.19)	1.93 (0.17)	1.9 (0.23)	1.94 (0.15)	1.71 (0.18)	2.28 (0.26)
Fe	45.86 (8.89)	12.64 (3.03)	11.42 (2.08)	9.07 (1.34)	14.42 (3.09)	5.1 (0.6)	7.41 (0.91)
Hg	1.78 (0.22)	1.06 (0.05)	1.05 (0.04)	1.02 (0.04)	1.03 (0.03)	1.18 (0.08)	1.06 (0.06)
K	211.14 (122.69)	21.38 (4.6)	18.77 (2.67)	14.49 (1.78)	18.44 (3.08)	18.12 (3.77)	20.92 (6.0)
Mn	17.15 (1.49)	2.74 (0.2)	2.56 (0.18)	2.18 (0.06)	2.44 (0.14)	2.27 (0.12)	2.6 (0.23)
Ni	3.42 (0.55)	1.6 (0.22)	1.57 (0.18)	1.21 (0.05)	1.45 (0.15)	1.31 (0.11)	1.55 (0.16)
Pb	2.1 (0.18)	2.75 (0.06)	2.72 (0.06)	2.69 (0.04)	2.71 (0.05)	2.77 (0.06)	2.73 (0.07)
P	11,7337.57 (13,428.73)	94,874.73 (7,329.33)	96,608.58 (6,822.27)	88,961.39 (4,931.65)	95,216.74 (7,162.93)	85,985.37 (5,494.47)	94,966.01 (6,449.53)
Se	2.38 (0.33)	1.09 (0.17)	0.94 (0.08)	0.94 (0.09)	0.93 (0.07)	2.57 (0.95)	1.5 (0.11)
S	14,722.77 (2,004.8)	13,084.37 (1,649.62)	12,851.85 (1,186.)	14,127.31 (1,296.12)	13,510.28 (1,119.52)	30,520.77 (6,416.25)	15,564.85 (1,844.26)
Sr	33.22 (6.28)	3.36 (0.44)	2.92 (0.37)	2.36 (0.12)	2.62 (0.26)	2.61 (0.22)	2.97 (0.27)
Zn	407.75 (54.5)	26.19 (6.84)	23.65 (6.35)	12.49 (2.8)	21.96 (6.02)	21.54 (6.61)	26.72 (7.64)

Distribution of 15 selected elements in the eye of 3 dpf wildtype zebrafish embryo are shown. Values are expressed in parts per million (ppm), roughly accounting for 5% of total eye volume. The mean values of measurements on three biological repeats are shown, followed by standard deviation in parentheses. RPE: retinal pigment epithelium, ONL/PR: outer nuclear layer/photo-receptor cell layer, INL: inner nuclear layer, IPL: inner plexiform layer, GCL: ganglion cell layer, LF: lens fibre, LE: lens epithelium.

**Table 2:** Tyrp1a/b knockdown reduced element amount in the RPE, but not in the retina.

Element	Structure	Control-MOs	Tyrp1a/b-MOs
Ba*	RPE	39.9 ± 12.55	24.73 ± 4.42
	Retina	27.93 ± 3.05	25.43 ± 4.45
	**BKG**	**7.95 ± 10.89**	**7.6 ± 10.41**
Br*	RPE	1.78 ± 0.47	1.08 ± 0.17
	Retina	1.24 ± 0.28	1.05 ± 0.15
	**BKG**	**0.36 ± 0.49**	**0.34 ± 0.47**
Ca*	RPE	651.43 ± 297.33	110.33 ± 14.94
	Retina	131.03 ± 23.99	137.51 ± 19.21
	**BKG**	**7.48 ± 10.24**	**7.09 ± 9.7**
Cu	RPE	3.57 ± 1.04	2.34 ± 0.65
	Retina	2.47 ± 0.64	2.54 ± 0.76
	**BKG**	**0.44 ± 0.6**	**0.42 ± 0.58**
Fe	RPE	25.47 ± 7.97	20.87 ± 9.18
	Retina	17.97 ± 6.11	21.14 ± 7.68
	**BKG**	**1.81 ± 2.54**	**1.25 ± 1.81**
K*	RPE	179.37 ± 77.93	84.6 ± 5.38
	Retina	88.23 ± 13.46	77.08 ± 5.89
	**BKG**	**20.11 ± 27.61**	**16.77 ± 23.04**
Mn*	RPE	6.68 ± 2.27	3.12 ± 0.42
	Retina	3.5 ± 0.8	2.99 ± 0.54
	**BKG**	**0.83 ± 1.14**	**0.8 ± 1.1**
Ni	RPE	3 ± 1.62	1.82 ± 0.56
	Retina	2.08 ± 0.4	1.99 ± 0.84
	**BKG**	**0.49 ± 0.67**	**0.48 ± 0.66**
Pb	RPE	3.46 ± 1.09	2.27 ± 0.45
	Retina	2.44 ± 0.34	2.25 ± 0.49
	**BKG**	**0.71 ± 0.97**	**0.68 ± 0.93**
P	RPE	26,858.17 ± 10,864	22,113.09 ± 4,789.48
	Retina	22,112.33 ± 5,423.21	23,004.26 ± 4,508.95
	**BKG**	**6,731.17 ± 9,246.91**	**7,700.35 ± 10,570.27**
Se*	RPE	1.62 ± 0.34	0.97 ± 0.07
	Retina	1 ± 0.13	0.93 ± 0.11
	**BKG**	**0.22 ± 0.31**	**0.22 ± 0.31**
S	RPE	5,654 ± 2,680.12	4,351.83 ± 981.91
	Retina	4,360.32 ± 1,275.18	4,584.08 ± 1,005.97
	**BKG**	**1,241.11 ± 1,708.96**	**1,455.61 ± 2,001.29**
Sr*	RPE	5.37 ± 1.92	2.58 ± 0.33
	Retina	2.72 ± 0.23	2.53 ± 0.33
	**BKG**	**0.78 ± 1.06**	**0.75 ± 1.02**
Zn*	RPE	102.28 ± 39.58	21.86 ± 4.54
	Retina	22.71 ± 4.35	24.93 ± 6.71
	**BKG**	**0.51 ± 0.7**	**0.55 ± 0.76**

The abundance of 14 selected elements in the RPE and the retina at 2 dpf was examined for embryos injected with either control-MOs (*n* = 4 embryos) or *tyrp1a/b*-MOs (*n* = 5 embryos). Values are expressed in parts per million (ppm). For each determination from control-MO and *tyrp1a/b*-MO injected embryos, the mean values of biological replicates are shown with standard deviation. As shown in Fig. 2D, elements with significant difference between embryos injected with control-MOs and *tyrp1a/b*-MOs are designated with * (Mann-Whitney *U* test; *U* = 0, *p* = 0.0158 for Ba, Ca, K, Mn, Sr and Zn; *U* = 1, *p* = 0.0365 for Br; *U* = 0, *p* = 0.0194 for Se). BKG: background measured outside of embryos (ppm).
