# Supplementary material for: Melanosomes in pigmented epithelia maintain eye lens transparency during zebrafish embryonic development
Source: Sci Rep. 2016 May 4;6:25046. doi: 10.1038/srep25046 (PMC4855227; doi:10.1038/srep25046)
Supplement: Supplementary Information [file srep25046-s1.pdf]

Melanosomes in pigmented epithelia maintain eye lens transparency during zebrafish embryonic development

Masanari Takamiya[1, 5], Feng Xu[2, 5], Heikki Suhonen[3, 6], Victor Gourain[1], Lixin Yang[1], Nga Yu Ho[1], Lukas Helfen[2, 3], Anne Schröck[1], Christelle Etard[1], Clemens Grabher[1], Sepand Rastegar[1], Günther Schlunck[4], Thomas Reinhard[4], Tilo Baumbach[2], Uwe Strähle[1]

Affiliations:

[1]Institute of Toxicology and Genetics, Karlsruhe Institute of Technology (KIT), Postfach 3640, 76021 Karlsruhe, Germany

[2]Institute for Photon Science and Synchrotron Radiation (IPS), Karlsruhe Institute of Technology (KIT), 76021 Karlsruhe, Germany

[3]European Synchrotron Radiation Facility, 38043 Grenoble, France

[4]Eye Center, Freiburg University Medical Center, Killianstr. 5, 79106 Freiburg, Germany

[5]equal contribution

[6]Current address: University of Helsinki, Department of Physics, 00560 Helsinki, Finland

Correspondence to Uwe Strähle

Supplemental materials

Figure S1 Zebrafish *unc45b* homozygous mutants show lens cataract

(A-B) Analysis of lens phenotype at 4 dpf by confocal reflection imaging of living wildtype embryos (WT, A) and homozygous *unc45b* mutants (*unc45b-/-*, B) raised in fish water. Anterior chamber is oriented to left. co: cornea. le: lens epithelium. The intensity of reflection is colour-coded as shown in the panel A. Abnormal lens reflections were observed with *unc45b* homozygous mutant embryos (arrows, B). Scale bar: 50 µm. (C-D) Lenticular reflection profiles as a function of distance from the anterior edge of the lens epithelium (le) toward the posterior

end of the lens are shown for WT (C) and *unc45b-/-* mutants (D). The intensity of reflection is shown in an arbitrary unit (AU). The number of examined individuals for each group is shown in the upper right corner. Profiles from individual embryos were overlaid. (E) *unc45b-/-* mutants (n=15 embryos) show significantly increased lens reflection compared to WT (n=23 embryos; Welch two sample t-test; t=-1.937, ***p=2.61 x10^{-11}). (F) RPE pigmentation was quantified by measuring transmission light through the eyes of WT embryos (n=25 embryos), *unc45b-/-* mutant embryos (n=10 embryos) and *albino* (*slc45a2*) homozygous embryos (n=69 embryos). One-way ANOVA revealed significant differences among three genotypes (F[2, 66]=119, p<2.2 x10^{-16}). Significant differences were observed with *albino* mutants in comparison to WT or *unc45b-/-* mutants (Tukey HSD test; ***p<2.2 x10^{-16}). No significant change was observed with the pigmentation status of RPE between wildtype and *unc45b* mutant eyes (Tukey HSD test, p=0.667).

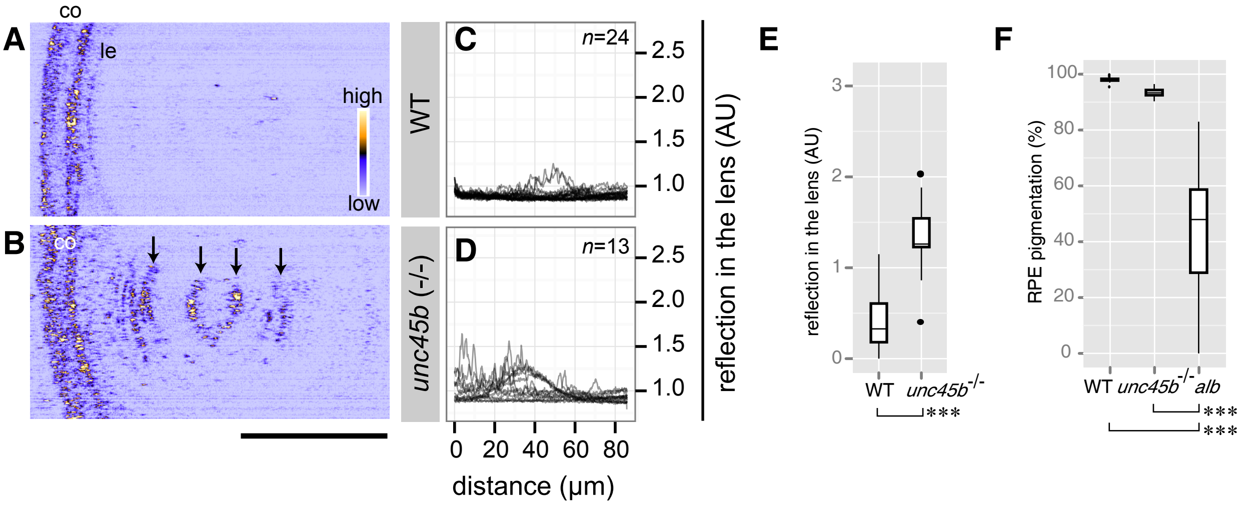

Figure S1 Zebrafish *unc45b* homozygous mutants show lens cataract

Table S1 Composition of positive ions in the fish water.

elements (ppb)	fish water
Li	0.21
B	9.75
Na	14,967.31
Mg	2,113.34
K	853.06
Ca	659.19
Ti	0.31
V	0.38
Cr	1.64
Mn	0.20
Fe	3.77
Co	0.11
Ni	1.22
Cu	0.55
Zn	n.d.
As	0.26
Rb	0.11
Sr	16.98
Mo	14.16
Ag	0.08
Cd	0.03
W	3.24
Re	*0.25
Hg	*0.15
Tl	0.08
Pb	n.d.

Raw measurement values (mean values calculated from two dilutions) were adjusted for errors through certified reference waters (SRM1643e and TMDA-51.3), except the values for Re and Hg (indicated by *). Elements below detection levels are indicated by n.d.. All values are expressed in µg/L (ppb).
